# The Impact of Intradialytic Exercise on Activities of Daily Living and Physical Function in Hospitalized Hemodialysis Patients: A Study of Efficacy and Safety

**DOI:** 10.31662/jmaj.2024-0349

**Published:** 2025-06-06

**Authors:** Ren Takahashi, Hiroki Yabe, Hideaki Ishikawa, Takashi Hibino, Akio Suzumura, Tetsuya Yamada

**Affiliations:** 1 Department of Rehabilitation, Kaikoukai Josai Hospital, Nagoya, Japan; 2 Department of Physical Therapy, Seirei Christopher University, School of Rehabilitation, Hamamatsu, Japan; 3 Department of Nephrology, Kaikoukai Josai Hospital, Nagoya, Japan; 4 Department of Neurology, Kaikoukai Josai Hospital, Nagoya, Japan; 5 Dialysis Division, Kaikoukai Healthcare Group, Nagoya, Japan

**Keywords:** rehabilitation, intradialytic exercise, hospitalized patient, hemodialysis, activity daily living, physical function

## Abstract

**Introduction:**

This study aimed to examine the effects of intradialytic exercise (IDE) on improving activities of daily living (ADL) and physical function in hospitalized hemodialysis (HD) patients. The research question focused on improving outcomes in ADL and physical function.

**Methods:**

This study is a single-center, historical cohort study. Subjects were hospitalized HD patients undergoing rehabilitation between April 2017 and February 2023. Patients were divided into two groups: the IDE group, which received IDE, and the non-IDE group, which did not. The outcomes measured were Barthel Index (BI), grip strength, isometric knee extension strength (IKES), 10-meter walking speed (10MWS), and Short Physical Performance Battery (SPPB). Outcome measures were taken at admission and discharge, and changes were analyzed using a linear mixed model.

**Results:**

The study included 76 participants (IDE group: 13, non-IDE group: 63). The IDE group showed significant improvements in ΔBI (13.7 [0.96-26.38] points) and Δ10MWS (0.25 [0.05-0.45] m/sec) (p < 0.05). No significant differences were observed between the two groups in ΔGrip strength (2.10 [−0.40 to 4.60] kg), ΔIKES (7.40 [−2.20 to 17.02] %), or ΔSPPB (1.23 [−0.48 to 2.94] points) (p > 0.05). However, the IDE group showed significant pre-post improvements in grip strength (1.55 [1.46-1.65] kg) and SPPB (2.44 [1.34-3.55] points) (p < 0.05).

**Conclusions:**

IDE contributed to greater improvements in BI and 10MWS in HD patients. These findings suggest that adding IDE to inpatient rehabilitation may enhance functional recovery in this population.

## Introduction

The increasing need for rehabilitation in hospitalized hemodialysis (HD) patients, particularly regarding activities of daily living (ADLs) and physical functionality, is evident. Previous studies indicate a 26% hospitalization rate over an average of 2.7 years ^(1)^, with hospitalization often linked to declines in ADL and physical function ^(2)^. ADL impairment in hospitalized HD patients is a significant predictor of in-hospital mortality and discharge to long-term care facilities ^(3)^. Therefore, enhancing ADL and physical function through targeted rehabilitation programs is crucial.

The effectiveness of rehabilitation in hospitalized HD patients is notably lower compared to non-HD patients, especially in ADL and physical function. Previous studies consistently demonstrate less ADL improvement in HD patients undergoing inpatient rehabilitation ^(4)^. A key factor contributing to this reduced effectiveness is the limited rehabilitation time available on HD treatment days ^(5)^. HD sessions, which last three to five hours, make it challenging to allocate sufficient rehabilitation time. Furthermore, fatigue ^(6)^, increased myocardial workload ^(7)^, and arrhythmias ^(8)^ complicate the scheduling of rehabilitation.

Intradialytic exercise (IDE) offers a promising strategy to enhance rehabilitation on HD days. Recent meta-analyses have demonstrated the positive impact of IDE on physical function and quality of life ^(9)^, leading to its recommendation in the Clinical Practice Guideline for Renal Rehabilitation as a safe and effective intervention for HD patients ^(10)^. Additionally, multiple studies confirm the safety of exercise therapy during HD sessions ^(11)^. Hospitalized HD patients are often limited in rehabilitation and physical activity due to HD treatment, and IDE may provide an opportunity to allow time for rehabilitation. However, most research on IDE efficacy and safety targets outpatient HD patients, with limited data on hospitalized HD patients.

The aim of this study is to provide empirical evidence regarding the efficacy and safety of implementing IDE for hospitalized HD patients. The study hypothesizes that IDE can significantly enhance improvements in ADL and physical function by providing additional time for rehabilitation.

## Materials and Methods

This study was a single-center, retrospective cohort study. Data were retrospectively extracted from a clinical database of hospitalized HD patients who underwent rehabilitation at Kaikoukai Josai Hospital. Data measured at two time points (admission and discharge) were used for secondary analysis.

The subjects were hospitalized HD patients who were admitted to the Community Care Unit from April 2017 to February 2023 and received subacute rehabilitation. The Community Care Unit is a ward where rehabilitation, medical care, and nursing care are provided to patients who have completed acute care and are ready to be discharged to their homes or nursing homes.

Inclusion criteria were: (1) patients who underwent inpatient rehabilitation, and (2) patients indicated for rehabilitation as defined by renal rehabilitation guidelines. Exclusion criteria were set for patients who could not appropriately perform IDE based on rehabilitation indications, including: (1) active physical or mental illness requiring acute medical management, (2) serious complications such as heart failure, severe infections, malignant tumors that preclude rehabilitation or necessitate discontinuation, (3) patients who were transferred to other hospitals or died during the rehabilitation period, (4) patients with cognitive decline (Hasegawa Dementia Scale - Revised [HDS-R] < 20), (5) patients whose IDE was judged by the attending physician to be infeasible, and (6) patients who did not consent to the study.

The study participants were stratified into two distinct cohorts: the IDE group, which received IDE during their inpatient rehabilitation, and the non-IDE group, which did not. The criteria for IDE application were based on the judgment of the attending physician and included patients who were stable on dialysis treatment, had sufficient cognitive function to understand the content of IDE, and had given their consent for IDE implementation. The initiation of IDE was determined collectively by the patient’s physical therapist (PT), occupational therapist (OT), and attending physicians, following the institution-wide adoption of IDE in April 2020. Patients who either did not actively seek IDE or had undergone rehabilitation prior to this implementation (i.e., before April 2020) were assigned to the non-IDE group.

This study was conducted in accordance with the principles of the Declaration of Helsinki. Approval was granted by the Ethical Committee of Seirei Christopher University (22049) and Nagoya Kyoritsu Hospital (K141-01). Informed consent for the study was secured by providing the option to opt out. Patients were informed in writing at the hospital and on the hospital website about how to decline participation in the study.

### Rehabilitation protocol

Rehabilitation was initiated following an assessment by the attending physician, who evaluated the necessity and prescribed appropriate interventions for patients hospitalized due to musculoskeletal, respiratory, cerebrovascular diseases, or disuse syndrome. A multidisciplinary team, including at least one PT, OT, or speech-language-hearing therapist (ST), provided therapy. Patients underwent physical or occupational therapy six days a week, excluding Sundays, with speech-language therapy added as needed. Rehabilitation was provided on both HD and non-HD days. The criteria for discontinuation followed the Guidelines for Renal Rehabilitation, with therapy halted in cases of contraindications, physician decisions, or at the patient’s request. On non-HD days, rehabilitation sessions were conducted in the rehabilitation room for 20 to 60 minutes, one to two times per day for both the IDE and non-IDE groups. These sessions included joint range of motion exercises, balance training, aerobic and resistance exercises, and ADL training aimed at improving and maintaining ADLs, with the long-term goal of facilitating home discharge. The duration, frequency, and content of rehabilitation were individualized by therapists based on the patient’s functional abilities. On HD days, rehabilitation for the non-IDE group was similar to that on non-HD days, lasting 20 to 40 minutes per session, once a day, either before or after HD. The IDE group received IDE in addition to the standard rehabilitation provided to the non-IDE group. The discharge date for HD patients was determined during a multidisciplinary team conference, involving the physician, nurse, PT or OT, medical social worker, and family members. A discharge evaluation was typically performed by the PT or OT one to three days before the planned discharge.

### IDE protocol

IDE was administered for 40 minutes per session, once daily, starting 30 minutes post-initiation of HD and within 90 minutes. The introduction of IDE was initiated after confirming the patient’s circulatory stability during HD from the time of admission, while also considering the progression of their rehabilitation. The IDE protocol was based on methods outlined in the clinical practice Guidelines for Renal Rehabilitation to enhance physical function ^(10)^. Stretching exercises included 20-second intervals of foot plantar flexion, dorsiflexion, rotation, straight leg raises, hip flexion, and hip abduction. Resistance exercises such as straight leg raises, hip abduction, hip flexion, and kicks were performed with elastic bands (TheraBand Resistance Band Loops, THERABAND, OH, USA), adjusted to the patient’s preferences and abilities. IDE was conducted as supervised exercise therapy by a PT. The intensity of resistance training was regulated to a Borg scale rating of 13 points. This rating of perceived exertion is a common index widely used in renal rehabilitation guidelines ^(10)^. The Borg scale was adjusted at the beginning of each session by the length and tension of the THERA BAND.

### Data collection

Patient characteristics included age, sex, body mass index and Geriatric Nutritional Risk Index (GNRI), rehabilitation time, and blood data (albumin, blood urea nitrogen, creatinine [Cre], sodium [Na], potassium [K], chlorine, phosphorus, calcium, estimated glomerular filtration rate, C-reactive protein [CRP], hemoglobin). Other data collected included HDS-R, comorbidities (diabetes mellitus, cancer, ischemic heart disease, hypertension, cerebrovascular disease, arteriosclerosis obliterans, valvular disease), Conditions requiring HD treatment (Diabetic nephropathy, Nephrosclerosis, Chronic glomerulonephritis, Other, Unknown), the reason for rehabilitation (Orthopedic, Pulmonary, Neurologic, Deconditioning), dialysis duration, dialysis time, Kt/V, percentage Cre generation rate (%CGR), and normalized protein catabolic rate (nPCR).

Outcomes included ADL, physical function, and length of hospital stay. Rehabilitation time was defined as the rehabilitation time per day and was calculated by collecting the total rehabilitation time, including PT, OT, and ST during the hospitalization period and dividing it by the number of hospital days. The calculation formula is:

Rehabilitation time (min/day) = Total hospitalization rehabilitation time (min) / length of hospital stay (days)

Data on ADL and physical function were collected at two points: upon admission and discharge, with the amount of change pre- and post-discharge calculated. ADL was assessed by the Barthel Index (BI) ^(12)^, which is a reliable and valid measure of ADL in HD patients ^(13)^. In this study, BI Gain and BI efficiency were calculated using the following formulas:

BI Gain = Discharge BI - Admission BI

BI efficiency = BI Gain / Length of hospital stay

Physical function was measured through grip strength, Isometric Knee Extension Strength (IKES), 10-meter walking speed (10MWS), Short Physical Performance Battery (SPPB), and Timed Up and Go (TUG) test at admission and discharge. Grip strength was measured using a grip strength meter (Grip-D digital grip strength meter, Takei Seisakusho Co., Ltd.) with the upper limbs in the extended position and, whenever position, in a standing position ^(14)^. For patients unable to hold a standing position, measurements were taken while seated. IKES was measured using a handheld dynamometer (Mobie, Sakaimed, Japan) in a seated position. The strap was fixed to a chair ensuring that isometric contraction could be performed with the knee at 90° during extension ^(15)^. Body weight ratio was calculated using dry weight at admission or discharge. Grip strength and IKES were calculated using the maximum left and right values. SPPB was evaluated on a 12-point scale, assessing three items: standing balance test, walking test, and chair standing test ^(16)^. SPPB in HD patients is an important indicator of overall physical function, as it has been associated with life expectancy and risk of hospitalization in HD patients ^(17)^. The 10MWS was measured by walking at maximum speed along a 14-meter straight walking path, including auxiliary paths (2 m each), and the time required to walk 10 meters was measured with a stopwatch. To measure TUG, participant was instructed to rise from a standard armchair, walk 3 meters as quickly and safely as possible, turn around at a cone placed by the researcher, walk back, and sit down. The timing began when the participant’s buttocks lifted from the seat and stopped when they were fully seated with their back against the backrest ^(16)^. In addition, the reference values of grip strength ^(18)^, IKES ^(19)^, 10MWS ^(18)^, SPPB ^(20), (21)^, TUG ^(20), (21)^, and BI ^(22)^ are listed in Table 1.

**Table 1.** Patient Characteristics.

		All group	Non - IDE group	IDE group	p-value
		n=76	n=63	n=13	
Age (year)		75.5 (9.31)	75.0 (1.2)	77.5 (2.8)	0.42
Sex [men, (n)%]		48 (63.2)	41 (65.1)	7 (53.8)	0.53
BMI (kg/m^2^)		21.4 (4.2)	22 (0.8)	21 (0.9)	0.42
GNRI		81.6 (9.1)	82.4 (1.2)	80.3 (2.4)	0.44
Prevalence of malnutrition (GNRI < 91.2) [(n)%]		58 (87.9)	45 (84.9)	13 (100)	0.12
Rehabilitation time (min/day)	All	39.1 (1.9)	37.0 (1.9)	49.0 (5.6)	0.01
	HD days	40.9 (2.0)	38.8 (1.8)	51.7 (6.8)	0.01
	Non-HD days	47.6 (2.5)	46.4 (2.7)	53.0 (6.3)	0.31
HDS-R (points)		25.7 (3.2)	25.8 (0.38)	25.1 (0.78)	0.42
Laboratory Data	
Alb (g/dL)		2.9 (0.4)	3.0 (0.1)	2.8 (0.2)	0.41
BUN (mg/dL)		41.9 (15.2)	42.1 (1.6)	40.8 (6.6)	0.85
Cre (mg/dL)		6.9 (2.1)	7.2 (0.3)	5.9 (0.5)	0.02
Na (mEq/L)		134.7 (3.4)	135.1 (0.4)	133.1 (0.9)	0.05
K (mEq/L)		3.8 (0.5)	3.9 (0.1)	3.5 (0.1)	0.04
Cl (mEq/L)		101.5 (3.8)	101.9 (0.5)	99.6 (1.1)	0.07
P (mg/dL)		3.7 (1.2)	3.8 (0.2)	3.3 (0.3)	0.11
Ca (mg/dL)		9.1 (0.9)	9.1 (0.1)	9.2 (0.2)	0.71
eGFR (mL/min/1.73m^2^)		6.8 (2.5)	6.5 (0.3)	8.1 (0.9)	0.11
CRP (mg/dL)		0.89 (1.24)	0.96 (0.17)	0.72 (0.45)	0.60
Hb (g/dL)		10.3 (1.3)	10.3 (0.2)	10.3 (0.4)	0.91
INT-PTH		132.3 (116.0)	136.9 (18.1)	126.7 (41.7)	0.83
Hemodialysis duration (months)		87.2 (88.8)	91.2 (13.2)	55.5 (21.7)	0.16
Hemodialysis Time (min)		229.0 (22.4)	230 (2.7)	223.8 (7.3)	0.43
Kt/v		1.35 (0.32)	1.36 (0.04)	1.33 (0.09)	0.77
%CGR (%)		68.3 (25.6)	69.2 (3)	63.6 (9.3)	0.57
nPCR (g/kg/day)		0.66 (0.15)	0.65 (0.02)	0.70 (0.06)	0.43
Reason for rehabilitation [Yes, n(%)]	
Orthopedic		53 (69.7)	44 (69.8)	9 (69.2)	0.62
Pulmonary		10 (13.2)	6 (9.5)	0 (0.0)
Neurologic		6 (7.9)	8 (12.7)	2 (15.4)
Deconditioning		7 (9.2)	5 (7.9)	2 (15.4)
Conditions requiring HD treatment [Yes, n(%)]	
Diabetic nephropathy		27 (35.5)	21 (33.3)	6 (46.2)	0.24
Nephrosclerosis		10 (13.2)	9 (14.3)	1 (7.7)
Chronic glomerulonephritis		2 (2.6)	1 (1.6)	1 (7.7)
Other		11 (14.5)	8 (12.7)	3 (23.1)
Unknown		26 (34.2)	24 (38.1)	2 (15.4)
Comorbidity [Yes, (%)]	
Diabetes mellitus		47 (61.8)	39 (61.9)	8 (61.5)	1.00
Cancer		8 (10.5)	7 (11.1)	1 (7.7)	1.00
Ischemic heart disease		29 (38.2)	25 (39.7)	4 (30.8)	0.76
Hypertension		30 (39.5)	23 (36.5)	7 (53.8)	0.35
Cerebrovascular disease		21 (27.6)	19 (30.2)	2 (15.4)	0.50
Arteriosclerosis obliterans		15 (19.7)	14 (22.2)	1 (7.7)	0.44
Valvular Heart Disease		4 (5.3)	3 (4.8)	1 (7.7)	0.54
Admission Physical function	Reference Value
Grip strength (kg)	Men: 28, Women 18	15.4 (5.3)	15.4 (0.8)	14.8 (1.5)	0.71
IKES (%)	40	26.4 (9.5)	25.76 (1.2)	28.76 (2.7)	0.38
10MWS (m/s)	1.0	0.66 (0.27)	0.65 (0.05)	0.72 (0.1)	0.52
SPPB (points)	9	2.8 (3.5)	2.7 (0.3)	3.3 (0.9)	0.55
TUG (sec)	13.5	28.5 (21.0)	28.5 (1.5)	27.7 (5.3)	0.88
Barthel Index (points)	75	53.9 (21.0)	55.7 (2.9)	50.8 (5.4)	0.43

Alb, albumin; BUN, blood urea nitrogen; BMI, Body mass index; Cre, creatinine; Ca, calcium; eGFR, estimated Glomerular Filtration Rate; Na, natrium; K, kalium; P, phosphorus; CRP, C-reactive protein; Cl, chlorine; Hb, hemoglobin; HDS-R, Hasegawa Dementia Scale-Revised; GNRI, Geriatric Nutritional Risk Index; IKES, Isometric Knee Extension Strength; 10MWS, 10M-Walking Speed; SPPB, Short Physical Performance Battery; TUG, Timed Up and Go test; The prevalence of malnutrition was defined as patients with a GNRI of less than 91.2. Since there were 10 cases with missing GNRI values, it was calculated for 66 cases.

To evaluate IDE safety, systolic blood pressure (SBP), diastolic blood pressure (DBP), and heart rate (HR) were monitored hourly during HD. Mean arterial pressure (MAP), double product (DP), and intradialytic hypotension (IDH) rates were calculated. Data were collected during two periods: the non-IDE Period (from admission to the start of IDE) and the IDE Period (from the start of IDE to discharge). Detailed data handling methods are described in Supplemental Text 1.

### Statistical analysis

Missing data were imputed using the multiple imputation method, generating 20 datasets for analysis. Categorical variables were expressed as percentages, while continuous variables were summarized as means with standard errors. Baseline characteristics were compared using Pearson’s χ^2^ test for categorical variables and unpaired t-tests for continuous variables. Outcome analysis employed a linear mixed model, allowing for both pre- and post-comparisons as well as group comparisons between the non-IDE (control) group and the IDE group. Fixed effects included time, group, and time-group interactions. This model calculated adjusted values differently from simple subtraction by accounting for fixed and variable effects. Specifically, the linear mixed model corrected for differences between groups and variations among individuals to derive results that reflected the overall trend. In addition, the “difference” did not correspond to a simple ΔIDE-Δ non-IDE because of the integration of observed data to obtain stable estimates.

In this study, three models were constructed to account for confounding factors influencing physical function and ADL improvements in hospitalized HD patients. Model 1 served as the baseline, unadjusted model. Model 2 adjusted for non-HD-related confounders, including age, gender, comorbidities (ischemic heart disease, hypertension, diabetes, cerebrovascular disease, arteriosclerosis obliterans), reason for rehabilitation, rehabilitation time, rehabilitation timing on HD days, HDS-R (cognitive function), GNRI (nutrition), and CRP (inflammation). Model 3 added HD-related factors to model 2, such as hemoglobin (anemia), HD time, Kt/V, nPCR, %CGR, HD duration, and conditions requiring HD treatment. Variables were chosen based on clinical hypotheses and prior research showing their relevance to physical function and ADL improvement.

The Akaike Information Criterion was used to compare model fits. Between-group differences were reported as means with 95% confidence intervals, while within-group differences were presented as means with standard errors.

The safety of IDE was assessed by comparing mean SBP, DBP, HR, MAP, and DP values at each 4-hour time point (Pre, 1 hour, 2 hours, 3 hours, 4 hours) during HD under non-IDE and IDE Periods. To account for potential non-linear hemodynamic changes, absolute values and changes (Δ) at each interval (Pre-1 hour, 1 hour-2 hours, 3 hours-4 hours) were analyzed using paired t-tests. Additionally, IDH incidence was compared pre- and post-test with paired t-tests.

Statistical analysis was performed using R Statistical Computing software ver. 4.0.3 (R Foundation for Statistical Computing, Vienna, Austria). The significance level was set at a risk rate of 5%.

## Results

**Figure 1.**
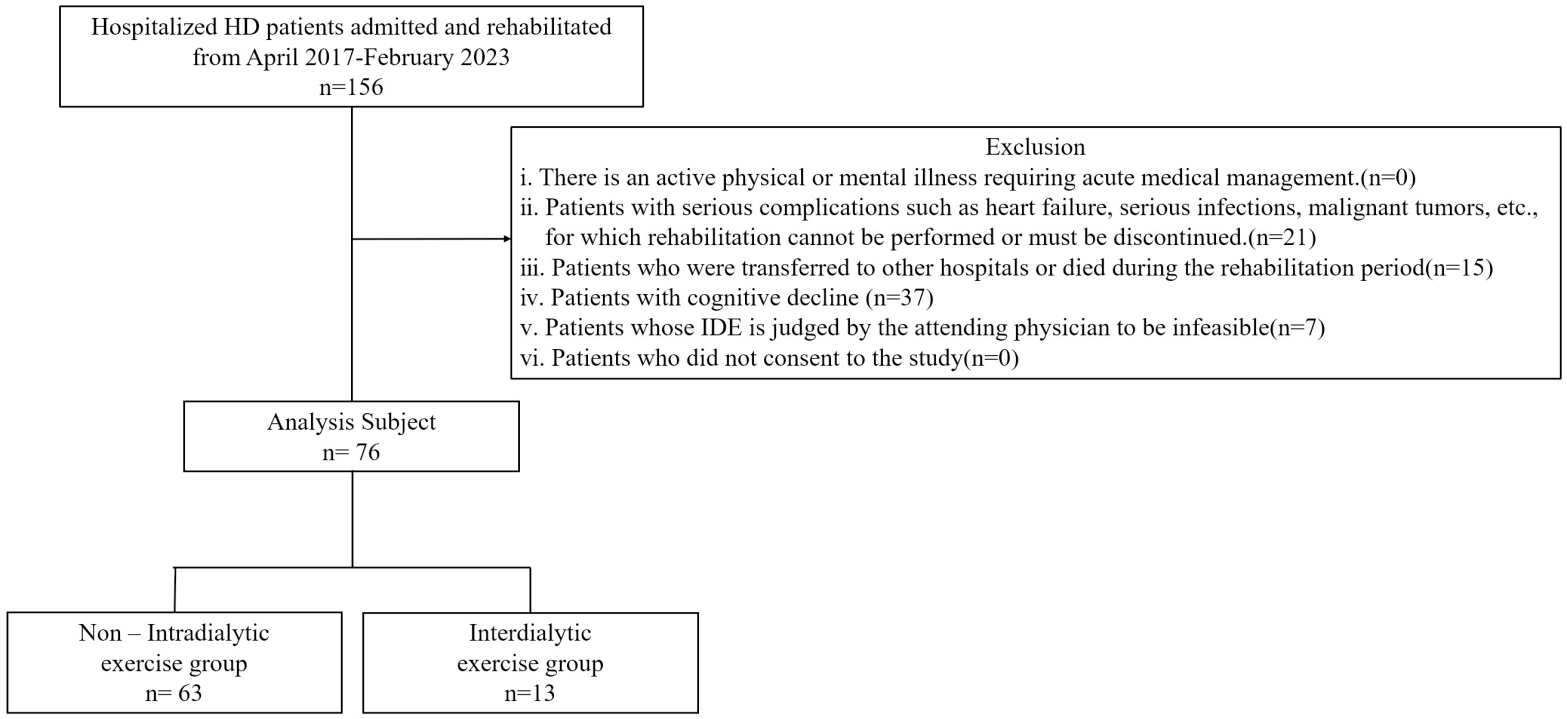
Subject flowchart.

Of the 156 hospitalized HD patients who underwent rehabilitation at Kaikoukai Josai Hospital between April 2017 and February 2023, 79 were excluded from the final analysis for various reasons. Ultimately, 76 patients were analyzed, with 63 in the non-IDE group (82.9%) and 13 in the IDE group (17.1%) (Figure 1).

Regarding patient background, there were no significant differences in age, sex, diabetes mellitus, or reason for rehabilitation. However, Cre, K, and Na were significantly lower in the IDE group (Table 1). The rehabilitation time was significantly higher in the IDE group compared to the non-IDE group (37.04 vs. 49.04 min/day, p = 0.097). While no difference was observed in rehabilitation time on non-HD days (47.55 vs. 53.02 min/day, p = 0.307), rehabilitation time on HD days was significantly greater in the IDE group (38.78 vs. 51.74 min/day, *p =* 0.009) (Table 2). The percentage of rehabilitation timing is detailed in Supplemental Table 1. There were no significant differences in physical function and ADL at admission (Table 1 and 2).

**Table 2.** Comparison of Rehabilitation Outcomes.

		All	Non-IDE group	IDE group	
		n=76	n=63	n=13	p-value
Length of hospital stays (days)	All	49.9 (1.8)	48.6 (1.9)	56.2 (4.0)	0.097
	HD days	21.3 (0.8)	20.7 (0.8)	23.9 (1.8)	0.105
	Non-HD days	21.6 (0.8)	21.1 (0.8)	24.2 (1.7)	0.111
Barthel Index Gain (points)		20.2 (1.6)	18.1 (1.4)	30.4 (5.4)	< 0.001
Barthel Index efficiency (points/days)		0.41 (0.05)	0.38 (0.05)	0.54 (0.08)	0.114
Discharge					
Grip strength (kg)		15.9 (6.3)	16.8 (1.9)	14.2 (2.7)	0.404
IKES (%)		28.7 (12.5)	28.1 (1)	32 (4.3)	0.368
10MWS (m/s)		0.70 (0.26)	0.69 (0.05)	0.77 (0.1)	0.516
SPPB (points)		4.9 (3.9)	4.8 (0.4)	5.2 (1.0)	0.695
TUG (sec)		20.4 (8.6)	20.3 (0.7)	20.9 (2.8)	0.819
Barthel Index (points)		69.2 (23.5)	65.6 (4.7)	81.2 (3.7)	0.010

IKES, Isometric Knee extension strength; 10MWS, 10-meter walking speed; TUG, Timed up and go test; SPPB, Short Physical Performance Battery.

There was no significant difference in the length of hospital stay (non-IDE group: 48.9 vs. IDE group: 56.2 days, p* =* 0.097).

Physical function and ADL at discharge were significantly higher in the IDE group only for BI (non-IDE group: 65.6 [4.7] points vs. IDE group: 81.2 [3.8] points, p *=* 0.010). In the pre-/post-comparison, grip strength (Δ1.55 [1.46-1.65] kg, p = 0.017) and SPPB (Δ2.44 [1.34-3.55] points, p *=* 0.006) were improved in the IDE group only. Both groups showed significant improvement in BI (non-IDE group: Δ16.26 [9.59-22.93], p < 0.001, IDE group: Δ28.90 [19.76-37.94] points, p < 0.001), but not in IKES (non-IDE group: *p =* 0.229, IDE group: p = 0.987) and 10MWS (non-IDE group: p* =* 0.279, IDE group: p *=* 0.073).

The results of the between-group comparison showed that ΔBI (13.67 [0.96-26.38] points, p* =* 0.047) and Δ10MWS (0.25 [0.05-0.45] m/sec, p *=* 0.036) were significantly greater in the IDE group than in the non-IDE group in model 3 adjusted for all confounding factors. ΔGrip strength (*p =* 0.115), ΔIKES (p* =* 0.149), ΔSPPB (p = 0.192), and ΔTUG (p = 0.739) did not differ between the groups (Table 3). The results of model 1 and model 2 are shown in Supplemental Table 2.

**Table 3.** Comparison of Physical Function and ADL Indices between Non-IDE Group and IDE Group.

	Non -IDE group (control)		IDE group		ΔIDE - ΔNon - IDE group	
	ΔPost - Pre (95CI)	p-value	ΔPost - Pre (95CI)	p-value	Difference (95CI)	p-value
Grip strength (kg)	0.85 (-0.27 to 1.97)	0.169	1.55 (1.46 to 1.65)*	0.017	2.10 (-0.40 to 4.60)	0.115
IKES (%)	-1.95 (-5.46 to 1.55)	0.299	0.07 (-0.14 to 0.29)	0.987	7.40 (-2.20 to 17.02)	0.149
10MWS (m/s)	0.04 (-0.03 to 0.13)	0.279	0.30 (0.12 to 0.47)	0.073	0.25 (0.05 to 0.45)*	0.036
SPPB (points)	1.06 (-0.003 to 2.116)	0.096	2.44 (1.34 to 3.55)*	0.006	1.23 (-0.48 to 2.94)	0.192
TUG (sec)	-10.80 (-19.49 to -2.12)*	0.035	-4.08 (-5.26 to -2.90)	0.975	-3.07 (-20.74 to 14.60)	0.739
Barthel Index (points)	16.26 (9.59 to 22.93)*	0.000	28.90 (19.76 to 37.94)*	0.000	13.67 (0.96 to 26.38)*	0.047

* p<0.05; Difference=(ΔIDE group - ΔNon -IDE group); TUG, Timed up and go test; SPPB, Short Physical Performance Battery; 95CI, 95% confidence Interval. Model 3, Adjusted for Age, sex, HDS-R, CRP, GNRI, comorbidities, reason for rehabilitation, rehabilitation time, rehabilitation timing on HD days, Hb, HD time, Kt/v, nPCR, %CGR, Hemodialysis duration, Hemodialysis causing disease.

**Figure 2.**
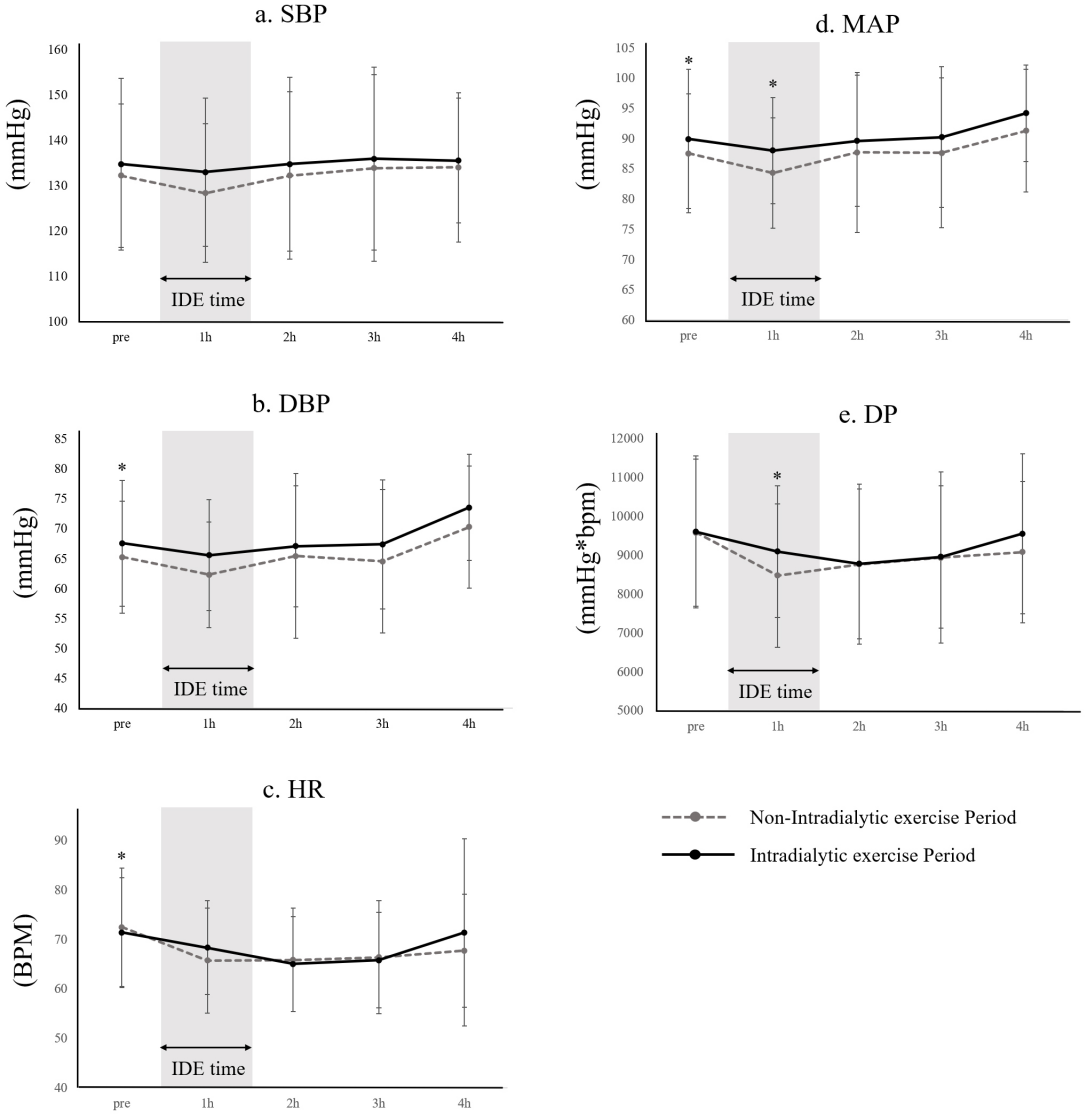
Comparison of blood pressure variability during HD in the IDE group. DBP: diastolic blood pressure; DP: double product; HR: heart rate; IDE: intradialytic exercise; IDE time: The period during which IDE was administered; MAP: mean arterial pressure; SBP: systolic blood pressure.

Pre- and post-IDE comparisons revealed significant differences. During the IDE Period, DBP (non-IDE: 65.3 [9.3] vs. IDE: 67.6 [10.5] mmHg) and MAP (non-IDE: 87.6 [9.9] vs. IDE: 90.0 [11.5] mmHg) at Pre were significantly higher than in the non-IDE Period. At 1h of the IDE Period, MAP (non-IDE: 84.4 [9.1] vs. IDE: 88.1 [8.8] mmHg) and DP (non-IDE: 8478.6 [1845.1] vs. IDE: 9096.4 [1700.9] mmHg*bpm) were significantly higher than in the non-IDE Period. HR (non-IDE: 72.5 [12.0] vs. IDE: 71.4 [11.1] bpm) at pre-HD was significantly lower than in the non-IDE period. In pre- and post-IDE changes, a significantly larger decrease in ΔDP at 1 hour-2 hours (non-IDE: [533.8]) was observed. No significant differences were identified in other parameters. The incidence of IDH showed no significant difference between periods (non-IDE: 6.2 [13.0]% vs. IDE: 9.7 [14.7]%) (Figure 2).

## Discussion

This retrospective study is the first to investigate the safety, feasibility, and efficacy of incorporating IDE into rehabilitation for enhancing physical function and ADL without compromising HD treatment. In this study, hospitalized HD patients who participated in IDE demonstrated significant improvements in the BI and 10MWS, with no adverse effects on vital signs or IDH frequency before or after IDE initiation.

The results of this study on the effectiveness and safety of IDE in hospitalized HD patients may contribute to improving the quality of rehabilitation for this population. While IDE has shown efficacy in outpatient HD patients, evidence in hospitalized settings remains scarce. A systematic review of exercise therapy for HD patients reported improvements in physical function, health-related quality of life, and HD-related symptoms ^(9)^. The Cochrane Review similarly concluded that exercise therapy enhances physical function in HD patients ^(23)^. However, most studies have primarily focused on outpatients, leaving hospitalized cases under-researched. Hospitalized HD patients, in particular, may have higher complication rates and be more prone to IDH and other HD-related effects during exercise therapy compared to outpatients ^(24)^. Therefore, further investigation into the effectiveness and safety of IDE in this population is essential.

The findings of this study revealed that the IDE group allocated more time to rehabilitation on HD days compared to the non-IDE group, achieving an equivalent volume of rehabilitation on both HD and non-HD days. Prior research has noted reduced rehabilitation time on HD days, resulting in limited functional improvements ^(4)^. These results suggest that incorporating IDE during HD days effectively ensures adequate rehabilitation time. However, as there was no significant difference in-hospital stay duration, further interventions targeting early discharge should be explored.

The IDE group demonstrated greater improvements in ΔBI and Δ10MWS speed compared to the non-IDE group. ADL and gait speed in HD patients serve as key prognostic factors and critical indices for improvement ^(25), (26)^. In this study, the observed changes of 13.67 points in BI and 0.25 m/sec in 10MWS are comparable to the Minimal Clinically Important Difference (MCID) reported in previous studies, which indicated a change of 9.25 points in BI ^(27)^ and 0.1 m/sec in 10MWS ^(28)^. Improvement over the MCID reflects a meaningful change in daily life and health status. In this study, the IDE group’s BI and 10MWS improvements likely reached clinically significant benefits based on the MCID. As for the impact on life prognosis, an improvement of 0.1 m/s in Δ10MWS ^(29)^ is reported to be associated with improved life expectancy, and an improvement of 0.25 m/s is considered clinically meaningful. On the other hand, the lower the BI value at discharge (cutoff value: 85 points), the poorer the prognosis ^(30)^, and an increase in the BI improvement rate due to IDE may contribute to improved prognosis. The increase in physical activity, likely attributed to extended rehabilitation sessions, may explain the observed improvements in both BI and 10MWS. HD patients, who typically undergo 4-hour HD treatments, often face challenges in leaving their beds and have limited opportunities for rehabilitation. Physical activity has been reported to decrease significantly on HD days ^(31)^. Nevertheless, improvements in ADL and gait speed have shown a dose-response relationship with rehabilitation ^(32)^. Thus, the inclusion of IDE likely contributed to the enhancements in ADL and 10MWS by ensuring sufficient physical activity on HD days.

Despite these improvements, no significant effect of IDE on muscle strength or balance function was observed. This contrasts with previous studies, which reported benefits of IDE on exercise tolerance, grip strength, IKES, SPPB, gait speed, and balance ^(9)^. The lack of improvement in IKES among hospitalized HD patients in this study may be attributed to the high risk of severe malnutrition, which could have hindered progress. Both groups in this study were categorized as being at high risk for severe malnutrition based on GNRI scores (non-IDE group: 82.4, IDE group: 80.3; cutoff: 82) ^(33)^. Furthermore, a study of ambulatory HD patients with a GNRI cutoff of 91.2 reported a prevalence of malnutrition of 39.1% ^(34)^. In contrast, in the present study, 87.9% of all patients were found to have malnutrition, indicating a higher prevalence. It has been reported that malnutrition may inhibit muscle protein synthesis and prevent muscle gains ^(35)^. Therefore, malnutrition is associated with decreased skeletal muscle mass and increased mortality, which may affect the efficacy of IDE in HD patients ^(36)^ Additionally, balance function is a complex motor task that requires neuromuscular coordination and functional control of the entire body, involving factors such as dynamic lower-body balance, agility, flexibility, and lower-limb muscle strength ^(37), (38)^. The lack of improvement in balance function may be due to the exercise regimen being confined to supine exercises. Future research should explore comprehensive rehabilitation strategies, including nutritional interventions and diverse exercise programs, to further enhance IKES and balance function in hospitalized HD patients.

IDE for hospitalized HD patients can be implemented without negatively impacting circulatory parameters during treatment. Therefore, we propose that IDE in hospitalized HD patients offers safety comparable to outpatient settings. One side effect of concern in the IDE group was the occurrence of IDH due to post-exercise hypotension following IDE. Since IDH can lead to interrupted HD treatment, it is important to prevent its occurrence. The results of our study indicated that circulatory dynamics post-IDE initiation showed a higher MAP one hour into HD and a decreased ΔDP within the first two hours, but HR did not change. These changes may be attributed to the fact that resistance training is intermittent due to its exercise characteristics, and this study employed an interval training format of three sets of eight repetitions with rest in between. Therefore, it is possible that the exercise was intermittent and did not lead to a continuous increase in HR. However, since this study focused on resistance training that incorporated extensive joint movements of the ankle and hip joints, it is possible that the muscle pumping action associated with lower extremity exercise may have promoted an increase in MAP by increasing venous return. There was a significant difference in the variability of HR and DBP before dialysis treatment between the two groups, but the variability was very small (HR: non-IDE: 72.5 [12.0] bpm vs. IDE: 71.4 [11.1] bpm; DBP: non-IDE: 65.3 [9.3] vs. IDE: 67.6 [10.5] mmHg), and the clinical impact was considered to be minimal. The results suggest that in this study, the prevalence of IDH did not change significantly pre- and post-IDE initiation, suggesting that it may contribute to the increase in SBP ^(39)^. We hypothesize that IDE helps stabilize MAP during the first hour of HD and mitigates rapid fluctuations in DP. However, it has been reported that physical activity ^(40)^, grip strength, muscle mass, heart disease, and autonomic nervous system disorders ^(41)^ can affect the maintenance of blood pressure during dialysis. Therefore, the exact mechanism is still unknown and requires further study, as unmeasured confounding remains.

This study has several limitations. First, it employs a historical cohort design, utilizing pre-existing database data. Consequently, unmeasured confounders may exist due to the exclusion of certain variables, potentially influencing ADL and physical function improvements. The amount of physical activity during hospitalization and the intensity and type of exercise during rehabilitation interventions may have an important impact on improving physical function and ADL. It has also been suggested that increased physical activity during hospitalization may increase the effectiveness of rehabilitation ^(42)^. In addition, psychological factors such as motivation for rehabilitation may also contribute to the effectiveness of rehabilitation. Rehabilitation time can be influenced by factors such as health status, psychological state, and dialysis schedule. However, this study did not fully explore these variations, leaving room for unmeasured confounding. Future research should address this limitation. Second, the study’s generalizability is restricted by the use of single-center data, limiting its applicability to other medical institutions. Additionally, this research focuses on a community-based comprehensive care ward with primarily subacute-stage patients. Future studies should include acute-stage patients and gather data from multiple centers to evaluate facility-specific factors comprehensively. The results of this study showed an effect size of 0.45 for BI in an IDE intervention focusing on resistance training in bed. With a two-tailed test, 80% power, and α = 0.05, the required sample size was estimated at 79 subjects per group. A future multicenter randomized controlled trial or prospective cohort study with this sample size is recommended. Third, rehabilitation outside the IDE program was customized to each patient’s pathology and discharge goals, making the individualized nature of rehabilitation a crucial factor to consider, as patient-specific differences may significantly affect outcomes. The individualization of rehabilitation reflects the diverse goals of patients (e.g., discharge with family assistance or living alone) and the corresponding rehabilitation content. However, in this study, exercise prescriptions adhered to the Safety Guidelines for Rehabilitation ^(10)^, ensuring that intensity remained within a safe and effective range. While tailored to individual rehabilitation goals, the prescribed intensity and volume were standardized within a range that ensures effectiveness and safety. Fourth, the sample size is small. IDE implementation in this study began in April 2020, and 43 patients were rehabilitated during 2023, of which 13 patients received IDE. Thus, the IDE uptake rate was 30.2%, which was low. The main reason for the low IDE induction rate is that IDE was performed exclusively by physiotherapists certified by the Japanese Society of Renal Rehabilitation as Registered Instructor of Renal Rehabilitation. However, only three physiotherapists at the facility had this certification, and the system for providing IDE was inadequate. This was one reason for the small number of IDE groups. When HD patients were prescribed rehabilitation, whether or not the physiotherapist treating the patient had a Registered Instructor of Renal Rehabilitation was completely random, so although there was a large element of randomness in whether patients were assigned to the IDE or non-IDE group, there may have been a potential selection bias. In other clinical situations, patients may refuse IDE. Detailed data on the reasons for patient refusal were not recorded in the study’s database, and the situation remains unclear. However, we estimate the following possible reasons for refusal. First, IDE in hospitalized HD patients was not generalized and safety concerns were strong among patients. In addition, low motivation for rehabilitation itself may have contributed to the low uptake rate. Although this study used a linear mixed model to account for sample size imbalance, it is possible that the estimation of fixed and random effects may be unstable when sample sizes are small, and that the results may not adequately reflect the population. Therefore, we believe it is important to increase the number of cases in future studies. Fifth, it is necessary to include cases of cognitive decline in the study. In this study, to exclude confounding effects of cognitive function as much as possible, patients with HDS-R scores of 20 or less were excluded from the study because they were considered to be in cognitive decline. However, for patients in the borderline range of mild cognitive decline (e.g., HDS-R score 18-19), IDE may be as safe as for patients with a score of 20. Therefore, future studies should examine the efficacy of IDE including patients with cognitive decline.

In conclusion, the inclusion of IDE in hospitalized HD patients was observed to extend rehabilitation duration and significantly enhance both ADL and walking speed. Moreover, IDE was safely administered without any adverse cardiovascular effects during HD sessions. Additionally, IDE effectively mitigated the reduction in MAP and lowered ΔDP one hour after the commencement of HD.

## Article Information

### Conflicts of Interest

None

### Acknowledgement

We would like to thank the patients who participated in this study and the hospital staff of the Kaikoukai Health Care Group for their tremendous cooperation during the study period.

### Author Contributions

Research idea and study design: Ren Takahashi, Hiroki Yabe; data acquisition: Ren Takahashi, Takashi Hibino, Hideaki Ishikawa; data analysis/interpretation: Ren Takahashi, Hiroki Yabe; statistical analysis: Ren Takahashi, Hiroki Yabe; supervision or mentorship: Hideaki Ishikawa, Akio Suzumura, Tetsuya Yamada. All authors read and approved the final manuscript.

### Approval by Institutional Review Board (IRB)

This study was performed in line with the principles of the Declaration of Helsinki. Approval was granted by the Ethical Committee of Seirei Christopher University (22049) and Nagoya Kyoritsu Hospital (K141-01).

### Data Availability

The data that support the findings of this study are available from the corresponding author upon reasonable request.

## Supplement

Supplemental Table 1

Supplemental Table 2

Supplemental Table 3

Supplemental Text 1
