# Supplementary material for: The Impact of Intradialytic Exercise on Activities of Daily Living and Physical Function in Hospitalized Hemodialysis Patients: A Study of Efficacy and Safety
Source: JMA J. 2025 Jun 6;8(3):834–45. doi: 10.31662/jmaj.2024-0349 (PMC12328902; doi:10.31662/jmaj.2024-0349)
Supplement: Supplemental Table 1 [file 2433-3298-8-3-0834-s001.pdf]

Supplemental Table 1. Percentage of timing of rehabilitation on HD days implementation for Non-IDE group and IDE group

		Non - IDE group n=63	IDE group n=13
Timing of rehabilitation on HD days [n(%)]	Post HD	40 (63.5)	-
	Pre HD	22 (34.9)	-
	Pre HD + Post HD	1 (1.6)	-
	IDE+Post HD	-	9 (69.7)
	IDE+Pre HD	-	3(23.1)
	IDE	-	1(7.7)

HD, Hemodialysis; IDE, Intradialytic exercise.
