# Supplementary material for: The Impact of Intradialytic Exercise on Activities of Daily Living and Physical Function in Hospitalized Hemodialysis Patients: A Study of Efficacy and Safety
Source: JMA J. 2025 Jun 6;8(3):834–45. doi: 10.31662/jmaj.2024-0349 (PMC12328902; doi:10.31662/jmaj.2024-0349)
Supplement: Supplemental Table 2 [file 2433-3298-8-3-0834-s002.pdf]

Supplemental Table 2. Results of group comparisons of all models of physical function and ADLs

	Model 1		Model 2		Model 3	
	Difference (95CI)	AIC	Difference (95CI)	AIC	Difference (95CI)	AIC
Grip strength (kg)	-0.62 (-1.77 to 3.00)	707.7	2.20 (-0.25 to 4.67)	369.7	2.10 (-0.40 to 4.60)	346.1
IKES (%)	3.81 (-2.90 to 2.90)	632.6	9.14 (0.19 to 18.10)	399	7.40 (-2.20 to 17.02)	370.6
10MWS (m/s)	0.04 (-0.12 to 0.20)	17.9	0.20 (-0.004 to 0.395)	48.3	0.25 (0.05 to 0.45)*	62.8
SPPB (points)	0.40 (-1.15 to 1.95)	303.2	0.94 (-0.76 to 2.64)	207.3	1.23 (-0.48 to 2.94)	212.4
TUG (sec)	1.61 (-12.17 to 15.39)	515.4	2.59 (-15.12 to 20.29)	353	-3.07 (-20.74 to 14.60)	334.7
Barthel Index	19.02 (7.09 to 30.96)*	1197.6	12.6 (0.79 to 24.33)*	552.4	13.67 (0.96 to 26.38)*	512.2

* $p<0.05$; Difference=(ΔIDE group - ΔNon - IDE group); TUG, Timed up and go test; SPPB, Short Physical Performance Battery; 95CI, 95% confidence Interval. Model 1, non-adjusted, Model 2, Adjusted for Age, sex, HDS-R, CRP, GNRI, presence of comorbidities, Reason for rehabilitation, rehabilitation time, rehabilitation timing on HD days. Model 3, Adjusted for Age, sex, HDS-R, CRP, BMI, GNRI, comorbidities, reason for rehabilitation, Hb, HD time, Kt/v, nPCR, %CGR, Hemodialysis duration, Hemodialysis causing disease.
