# Supplementary material for: The Impact of Intradialytic Exercise on Activities of Daily Living and Physical Function in Hospitalized Hemodialysis Patients: A Study of Efficacy and Safety
Source: JMA J. 2025 Jun 6;8(3):834–45. doi: 10.31662/jmaj.2024-0349 (PMC12328902; doi:10.31662/jmaj.2024-0349)
Supplement: Supplemental Table 3 [file 2433-3298-8-3-0834-s003.pdf]

Supplemental Table 3. Mean values and coefficients of variation of SBP, DBP, HR, MAP, and DP for non-IDE and IDE periods.

		Maximum value			Minimum value			all value		
		Pre-IDE	Post-IDE	p-value	Pre-IDE	Post-IDE	p-value	Pre-IDE	Post-IDE	p-value
SBP	0	156.33 (18.68)	164.77 (31.59)	0.43	110.25 (19.83)	109.00 (24.24)	0.89	132.20 (15.81)	134.72 (18.99)	0.11
	1	152.08 (17.87)	163.54 (23.25)	0.183	106.25 (21.52)	106.62 (21.22)	0.966	128.36 (15.34)	132.96 (16.37)	0.06
	2	157.17 (20.24)	167.23 (25.62)	0.29	109.92 (25.34)	109.46 (20.28)	0.961	132.24 (18.44)	134.74 (19.21)	0.34
	3	154.58 (20.82)	163.31 (22.61)	0.327	106.00 (27.52)	108.92 (21.34)	0.768	133.87 (20.58)	135.95 (20.22)	0.63
	4	165.88 (23.04)	168.78 (29.58)	0.826	108.50 (18.69)	105.33 (14.49)	0.7	134.06 (16.46)	135.52 (13.74)	0.59
	CV	6.65 (3.35)	6.76 (3.48)	0.937	10.47 (5.25)	10.78 (5.32)	0.887	4.80 (2.34)	5.84 (2.46)	0.29
DBP	0	77.67 (11.08)	86.38 (20.67)	0.207	54.42 (12.27)	54.46 (9.33)	0.992	65.28 (9.33)	67.59 (10.51)	< 0.05
	1	72.75 (12.26)	81.54 (11.42)	0.076	51.33 (9.04)	46.92 (14.77)	0.382	62.35 (8.81)	65.61 (9.25)	0.05
	2	85.08 (35.63)	82.85 (15.01)	0.837	50.42 (11.35)	51.31 (12.17)	0.852	65.48 (13.76)	67.12 (10.12)	0.79
	3	78.08 (15.93)	81.69 (15.00)	0.565	50.50 (14.82)	53.54 (13.16)	0.592	64.57 (12.00)	67.42 (10.78)	0.31
	4	89.25 (20.13)	96.56 (15.40)	0.411	53.88 (13.65)	55.56 (5.85)	0.741	70.31 (10.24)	73.60 (8.84)	0.17
	CV	12.28 (11.35)	9.90 (5.73)	0.51	11.79 (8.04)	13.75 (10.26)	0.602	7.17 (3.26)	5.69 (3.39)	0.279
HR	0	82.50 (15.65)	79.69 (14.19)	0.642	65.67 (12.17)	59.00 (13.58)	0.21	72.50 (12.00)	71.44 (11.11)	< 0.05
	1	74.83 (9.01)	78.85 (12.57)	0.372	58.83 (12.45)	59.92 (10.78)	0.817	65.76 (10.62)	68.34 (9.49)	0.09
	2	74.33 (10.02)	75.54 (10.83)	0.776	57.08 (11.53)	56.62 (8.87)	0.91	65.89 (10.48)	65.06 (9.61)	0.28
	3	74.50 (10.32)	76.85 (11.65)	0.6	59.17 (12.90)	56.54 (9.29)	0.562	66.42 (11.42)	65.84 (9.65)	0.50
	4	78.88 (12.38)	79.00 (12.74)	0.984	57.88 (9.39)	58.44 (8.92)	0.9	67.76 (11.41)	71.43 (18.91)	0.47
	CV	6.98 (7.57)	6.61 (3.74)	0.879	8.89 (4.01)	8.22 (6.65)	0.763	5.62 (3.90)	7.41 (5.61)	0.37
MAP	0	101.92 (10.79)	110.03 (21.06)	0.244	73.97 (13.03)	73.31 (12.64)	0.898	87.59 (9.85)	89.97 (11.51)	< 0.05
	1	97.39 (13.79)	107.74 (12.05)	0.057	70.36 (11.65)	69.00 (12.64)	0.783	84.36 (9.14)	88.06 (8.78)	< 0.05
	2	107.69 (28.85)	109.56 (14.89)	0.839	71.22 (13.81)	72.56 (12.85)	0.804	87.74 (13.21)	89.66 (10.84)	0.57
	3	102.47 (13.56)	106.49 (15.34)	0.496	69.72 (17.33)	73.85 (13.89)	0.516	87.67 (12.40)	90.26 (11.65)	0.43
	4	108.67 (16.18)	117.52 (17.40)	0.297	73.96 (9.56)	73.48 (7.32)	0.909	91.32 (10.15)	94.24 (8.00)	0.26
	CV	8.15 (6.17)	8.08 (4.28)	0.971	10.41 (6.43)	10.86 (5.32)	0.848	5.09 (2.55)	5.64 (2.85)	0.62
DP	0	11575.75 (2139.20)	12445.62 (2963.39)	0.412	7691.17 (1950.36)	7100.31 (2231.84)	0.49	9584.48 (1897.85)	9606.54 (1956.18)	0.73
	1	10417.17 (2041.10)	11926.69 (2407.45)	0.106	6756.75 (2270.97)	6798.31 (1734.06)	0.959	8478.58 (1845.06)	9096.38 (1700.92)	< 0.05
	2	10847.25 (2619.73)	11360.92 (2748.61)	0.638	6691.33 (1808.87)	6892.46 (1871.18)	0.787	8770.72 (2060.31)	8784.23 (1926.50)	0.89
	3	10811.67 (2020.58)	11553.62 (2146.43)	0.384	6914.08 (2469.01)	6618.77 (2139.01)	0.752	8941.40 (2201.45)	8959.12 (1835.93)	0.93
	4	11517.38 (1496.78)	12140.00 (2569.08)	0.558	6958.75 (2010.23)	6871.22 (1137.26)	0.912	9086.70 (1816.02)	9560.57 (2060.37)	0.30
	CV	9.65 (4.03)	11.05 (6.74)	0.539	14.59 (6.71)	12.97 (6.80)	0.556	8.42 (3.66)	9.78 (5.29)	0.467

SBP, systolic blood pressure; DBP, diastolic blood pressure; HR, Heart rate; MAP, mean arterial pressure, DP, Duble product; CV, Coefficient of Variation.
