# Supplementary material for: The Impact of Intradialytic Exercise on Activities of Daily Living and Physical Function in Hospitalized Hemodialysis Patients: A Study of Efficacy and Safety
Source: JMA J. 2025 Jun 6;8(3):834–45. doi: 10.31662/jmaj.2024-0349 (PMC12328902; doi:10.31662/jmaj.2024-0349)
Supplement: Supplemental Text 1 [file 2433-3298-8-3-0834-s004.pdf]

Supplemental Text 1. Detailed description of safety indicators for Intradialytic exercise

For systolic blood pressure (SBP), diastolic blood pressure (DBP), heart rate (HR), mean arterial pressure (MAP), and double product (DP), the mean, maximum, and minimum values of data at each time point from 1 to 4 hours of HD were collected. In addition, coefficients of variation were calculated for each maximum and minimum value, as well as for the mean of all data, using the values before dialysis treatment and from 1 to 4 hours. These data were added to Supplementary Table 3.

Intradialytic hypotension (IDH) was defined as SBP less than 90 mmHg during a single HD session[1]. The number of occurrences of IDH during each collection period was then recorded. The incidence of IDH was then calculated from the total number of HD cycles and the number of IDH occurrences using the following formula

IDH rate (%)=Number of IDH events (times)/Total Number of Dialysis Cycles (Times)

Refference

1. Flythe JE, Xue H, Lynch KE, et al. Association of mortality risk with various definitions of intradialytic hypotension. J Am Soc Nephrol. 2015;26(3):724-734.
